# Differences of immune disorders between Alzheimer’s disease and breast cancer based on transcriptional regulation

**DOI:** 10.1371/journal.pone.0180337

**Published:** 2017-07-18

**Authors:** Wei Kong, Xiaoyang Mou, Jin Deng, Benteng Di, Ruxing Zhong, Shuaiqun Wang, Yang Yang, Weiming Zeng

**Affiliations:** 1 College of Information Engineering, Shanghai Maritime University, Haigang Ave., Shanghai, P. R. China; 2 Department of Biochemistry, Rowan University and Guava Medicine, Glassboro, New Jersey, United States of America; 3 Department of Computer Science and Engineering, Shanghai Jiao Tong University, Shanghai, P. R. China; College of Bioinformatics Science and Technology, CHINA

## Abstract

Although chronic inflammation and immune disorders are of great importance to the pathogenesis of both dementia and cancer, the pathophysiological mechanisms are not clearly understood. In recent years, growing epidemiological evidence and meta-analysis data suggest an inverse association between Alzheimer’s disease (AD), which is the most common form of dementia, and cancer. It has been revealed that some common genes and biological processes play opposite roles in AD and cancer; however, the biological immune mechanism for the inverse association is not clearly defined. An unsupervised matrix decomposition two-stage bioinformatics procedure was adopted to investigate the opposite behaviors of the immune response in AD and breast cancer (BC) and to discover the underlying transcriptional regulatory mechanisms. Fast independent component analysis (FastICA) was applied to extract significant genes from AD and BC microarray gene expression data. Based on the extracted data, the shared transcription factors (TFs) from AD and BC were captured. Second, the network component analysis (NCA) algorithm in this study was presented to quantitatively deduce the TF activities and regulatory influences because quantitative dynamic regulatory information for TFs is not available via microarray techniques. Based on the NCA results and reconstructed transcriptional regulatory networks, inverse regulatory processes and some known innate immune responses were described in detail. Many of the shared TFs and their regulatory processes were found to be closely related to the adaptive immune response from dramatically different directions and to play crucial roles in both AD and BC pathogenesis. From the above findings, the opposing cellular behaviors demonstrate an invaluable opportunity to gain insights into the pathogenesis of these two types of diseases and to aid in developing new treatments.

## Introduction

Alzheimer's disease (AD), which is the most common form of dementia, is a progressively fatal neurodegenerative disorder characterized by irreversible cognitive and memory deterioration that inevitably leads to death. AD has been known for more than 100 years and affects more than 13% of people older than 65 and 43% older than 85. However, the genetic mechanism and pathogenesis of AD are still unclear. An increasing number of studies show that chronic inflammation and immunosenescence play an important role in the progression of AD [1, 2]. Cancer, another type of age-related disorder, is a major health problem today. The immune system has been shown to play a significant role in its pathogenesis, though the exact pathophysiological mechanism has not been clearly defined [3, 4].

In recent years, growing epidemiological evidence suggests that there is an inverse relationship between cancer and neurodegenerative diseases, particularly AD [5–8]. That is, people with AD have a decreased risk of cancer, while people with prevalent cancer have a lower risk of AD. For example, a prospective longitudinal study involving 5278 elderly people diagnosed and undiagnosed with dementia for a median of 12.5 years showed that cancer-specific mortality was inversely associated with AD and other types of dementia [9]. In their study, cancer was reported significantly less often in individuals with possible or probable AD (5.8%) or non-AD dementia (6.3%) than in those without dementia (26.5%). In their unadjusted Cox model, the risk of cancer-specific mortality was decreased in participants with AD (hazard ratio = 0.45) compared to patients with other non-AD dementia (hazard ratio = 0.62) and those without dementia (reference group). Another prospective cohort study that included 3020 white adults aged 65 years or older with means of 5.4 and 8.3 years for dementia and cancer, respectively, showed that the presence of AD had a reduced risk of future cancer hospitalization and that a history of cancer might decrease the risk of AD [6]. In contrast, no significant association has been found between cancer and the development of vascular dementia (VaD) [6, 10]. This view is also supported by the epidemiological and demographic analysis in the study by [11]; specifically, there was shown to be an inverse association between tumors and AD that was more evident in women and for endocrine-related tumors. Furthermore, many longitudinal cohort studies and meta-analyses have revealed that the inverse association between AD and cancer was not due to the earlier mortality of cancer or under-diagnosis or under-reporting of cognitive impairment because the patterns were present both before and after the diagnosis of each disease and in both survivors and non-survivors [6, 9]. Although these epidemiological studies and meta-analyses indicate that the rate of developing cancer might decrease the risk of AD over time and vice versa, the biological and pathophysiological mechanisms for the inverse association between these two diseases are not clear.

We know that the characteristic pathology in AD changes and increases neurotic and synaptic degeneration as well as neuronal cell death. Conversely, cancer is a process of unlimited cellular proliferation. This suggests that AD and cancer may share certain fundamental biological processes, particularly those favoring apoptosis and cell proliferation, such as metabolic dysregulation, oxidative stress, DNA damage/repair, aerobic glycolysis, inflammation and immunosenescence, which play critical but opposite roles in both diseases [12–16]. Some shared proteins, including p53, a typical stress response gene and tumor suppressor related to the development of cancer when it is inactivated [17–19], correlated with the accumulation of Amyloid beta(Aβ), which is a neuropathological hallmark of AD, and led to progressive degeneration and neuronal death when activated [20]. Pin1 is an intracellular signaling molecule that plays a key role in the cell cycle and cell signaling, regulation of transcription and splicing, and neuronal protein maintenance, including beta-amyloid and tau [21]. It has been reported that Pin1 is inversely expressed in AD and cancer [19, 22]. Some common biological processes and pathways, such as the Wnt signaling pathway, ubiquitin–proteasome system (UPS), cellular metabolism, elevated aerobic glycolysis and oxidative phosphorylation (OxPhos), have been revealed to play opposite roles in AD and cancer [11,12,14,16,17]. The ERK/MAPK pathway has also been found to be oppositely regulated between glioblastoma (GBM) and AD [23]. Particularly, estrogens have been shown to play an important role in operating mitochondrial activity, modulating growth and synaptic plasticity, reducing neuronal apoptosis and Aβ formation, and inducing tau protein synthesis, which could explain the inverse associations between female-related tumors and AD [11,15].

Furthermore, biochemical and neuropathological studies of AD brains provide clear evidence for an activation of inflammatory pathways [24]. Recent studies have shown that AD deterioration is closely correlated with immune processes through amyloid clearance deterioration, increase of amyloid deposition by activated inflammatory cytokines, and alteration of the receptor for advanced glycolytic end products [1,2,13]. Additionally, the contribution of the innate and adaptive immune systems is of great importance to the pathogenesis of cancer. However, the high-throughput microarray data are also limited to understanding whether and how the inverse association occurs in immune processes between these two types of diseases. Therefore, further study of the whole transcriptome may provide insight into the contribution of the immune system and the pathophysiological differences between AD and cancer.

The aim of our study is to identify inverse signaling pathways from the immune response mechanism between AD and cancer via a bioinformatics method. We first performed research on breast cancer (BC) because it is one of the most common cancers in the world. Because the survival rate is high, the public gene expression microarray datasets for BC are abundant.

To find certain fundamental biological processes and shared genes that are differentially expressed in both AD and breast cancer, fast independent component analysis (FastICA) [25], an efficient biclustering matrix factorization technique among various ICA methods, was applied to identify significant genes from the AD and BC microarray datasets. ICA has the ability to group genes in different meaningful biological processes so that the feature genes can be easily discovered from different signaling pathways. And its improved algorithms such as ICA-based penalized discriminant method were successfully used for tumor classification [26]. This method outperforms principal component analysis (PCA) and traditional clustering methods, such as k-mean, self-organizing maps (SOM) and hierarchical clustering in feature extraction, as well as the classification of gene expression datasets [27–29].

In general, it is known that transcriptional activity is often controlled by only a relatively small set of transcription factors in many biological systems. In light of the significant genes extracted by FastICA, the shared genes, especially the transcription factors (TFs), in AD and breast cancer can be captured. However, the hidden regulatory structures and TF activities on target genes (TGs) cannot be determined from microarray datasets. To overcome this shortcoming, network component analysis (NCA) developed by Liao et al. [30] was applied to determine the TF activities and regulatory influences on TGs in AD and BC.

## Methods

### Independent Component Analysis (ICA) in gene expression data

ICA is a high-order statistical and unsupervised algorithm that has been widely used in voice signal blind separation, array processing, image processing, and medical ICA is a high-order statistical and unsupervised algorithm that has been widely used in voice signal blind separation, array processing, image processing, and medical and biological signal analysis and has been recently successfully used in gene clustering, classification and pathway and biomarker identification. For gene expression datasets, ICA assumes that the gene expression data represent a linear combination of specific independent biological process; therefore, the ICA model can be written as

X=AS (1)

where ***X*** denotes *n*×*m* microarray gene expression data with *m* genes under *n* samples or conditions. *x*_*ij*_ in ***X*** is the gene expression level of the *j*-th gene in the *i*-th sample; ***A*** = [*a*_1_, *a*_2_, …, *a*_n_] are the *n*×*n* mixing matrix; and ***S*** denotes the *n*×*m* gene signature matrix or expression mode, the rows of which are statistically independent on one another. The gene expression matrix ***X*** is considered to be a linear combination of the independent specific biological process rows of ***S***.

The matrix relationship shows that the *i*-th row matrix ***A*** contained the weights with which the expression levels of *m* genes contribute to the *i*-th row of gene expression profile ***X***. The classification information for gene profile ***X*** is equivalent to the rows of ***A***. Additionally, each column of ***A*** contains the weights with which *s*_*n*_ contributes to all of the observations and corresponds to one row of ***S***. Therefore, if some columns in ***A*** are identified to match the class labels in matrix ***X***, their corresponding rows in ***S***, *s*_*n*_, must contain useful biological information for classification. The significant gene expression levels in *s*_*n*_ may be the feature genes related to disease regulatory pathways. To obtain the independent biological process ***S*** and mixing matrix ***A***, the demixing model can be expressed as

$$Y=WX\approx A^{\text{-}1}\cdot AS$$ (2)

where ***W*** is an *n*×*n* demixing matrix. FastICA, which was developed by Hyvärinen et al, and this package can be downloaded from http://research.ics.aalto.fi/ica/fastica/, is one of most efficient and popular ICA algorithms for obtaining ***Y*** and ***W*** [25]. In FastICA, maximizing negentropy is used as the contrast function to measure the non-Gaussianity so that the iterative components can be statistically independent, or as independent as possible. To estimate the negentropy of ***y***_i_ = ***wx***_*i*_, an approximation to identify the independent components is designed as follows:

$$J_{G}(w)={\left[E\{G(w^{T}x)\}-E\{G(v)\}\right]}^{2}$$ (3)

where ***G*** can be practically any non-quadratic function and is selected according to the probability density distribution of the output signal ***y***, E{·} denotes the expectation, and *v* is a Gaussian variable of zero mean and unit variance.

In our former studies, FastICA was successfully used to identify many significant genes and related pathways that play prominent roles in AD phenotypes as well as identify associations, such as inflammation and APP expression, in molecular biological experiments.

### Network Component Analysis (NCA) extracts transcription factor activities

NCA is a network structure-driven matrix decomposition method presented by Liao et al. [30] that can be used to deduce the quantitative TF activity (TFA) and regulatory control strength (CS) from gene expression data and connectivity networks; this analysis provides the qualitative regulatory information between TFs and their target genes (TGs). They approximate the relationship between TFA and CS by a log-linear model as follows:

$$\frac{E_{i}(t)}{E_{i}(t)}=\prod {\left(\frac{TFA_{j}(t)}{TFA_{j}(0)}\right)}^{CS_{ij}}$$ (4)

where ***E***_*i*_(*t*) is the expression level of gene *i*, TFA_*j*_(*t*) with *j* = 1, …; *L* is the activity level of TF *j*; *CS*_*ij*_ represents the control strength of TF *j* on gene *I*; and (*t*) and (0) designate condition *t* and the reference condition 0, respectively. Then, log-linear transformation is used to model this nonlinear system. NCA models the expression of a gene as a linear combination of the activity of each TF that controls the expression of the gene. The matrix form of Eq 4, after taking the logarithm, is shown as follows:

log[E]=[CS]log[TFA] (5)

where ***E*** is the *N*×*M* gene expression matrix of *N* genes in *M* samples, ***E***_*i*_ ***= E***_*i*_ (*t*)/***E***_*i*_ (0); ***TFA*** is the *L*×*M* matrix that denotes the activity of *L* TFs on *M* samples while ***TFA***_*i*_ ***= TFA***_*i*_ (*t*)/ ***TFA***_*i*_ (0). The matrix [***CS***] represents the *N*×*L* connectivity strength of *L* TFs on *N* TGs. This log-linearized form can be expressed in a canonical matrix form as:

[E]=[C][P] (6)

This matrix form denotes that gene expression is expressed as a linear combination of TFAs weighted by their control strengths. The element *c*_*ij*_ in matrix [***C***] is set to 0 if there is no prior regulatory information for gene *i* by TF *j*; otherwise, it is set to a nonzero number as an initial value. To guarantee a unique decomposition solution of [***E***], Liao et al. presented three uniqueness criteria for [***C***] and [***P***] that should be satisfied. To find the best solution for Eq 6, the following least-square algorithm is performed:

$$\min{\left\|\left[E]-[C][P\right]\right\|}^{2},s.t.C\in Z_{0}$$ (7)

where ***Z***_0_ is the topology induced by the network connectivity pattern. Then, the actual estimation of [***C***] and [***P***] is performed by a two-step alternating least-squares algorithm that exploits the biconvexity properties of linear decompositions (for specific details, see [30] by Liao et al. (2003)). The NCA toolbox for Matlab can be downloaded from http://www.seas.ucla.edu/liao_lab/downloads.html.

### The procedure of exploring the differences of regulatory activities

**Fig 1:**
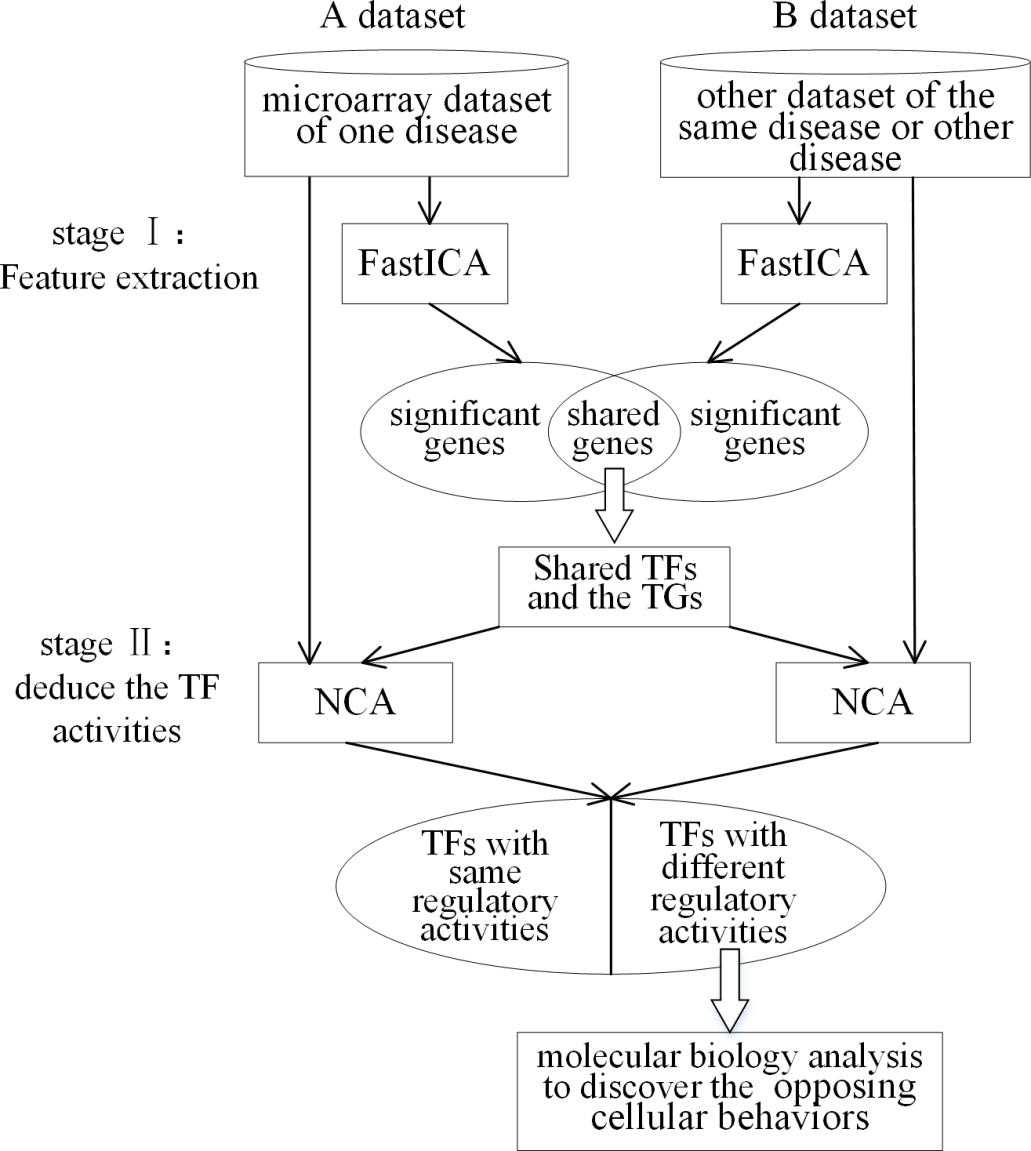
Structure flowchart of the proposed two-stage procedure. This flowchart shows the two-stage procedure in this study to detect the inverse transcriptional regulatory process of AD and BC. The first stage is to extract the feature genes of AD and BC datasets by FastICA, and the second stage is to deduce the regulatory activities and control strength of common TFs by NCA, then the TFs with inverse association will be selected for further study.

The structure flowchart of the proposed two-stage procedure for comparing the significantly cellular behaviors of two diseases is showed in Fig 1. In the first stage, significant genes of different diseases or datasets are extracted by using FastICA respectively, then the shared significant genes of these two diseases/datasets can be obtained; in the second stage, the common TFs out of the shared genes are found out to reconstruct the transcriptional regulatory network by NCA algorithm, with which the regulatory activities and control strength of the same TFs both in AD and BC can be discovered. The main target of this study is to investigate the inverse association between AD and BC, therefore, the molecular biological functions of the TFs with opposite activities are further studied.

In addition, another kind of experiments for one disease (AD or BC) on different datasets have been studied in our research to investigate the biological mechanism between different subtypes or grades in the same disease. The same two-stage procedure are performed on different datasets of AD or BC. According to the NCA results, the common and opposite TF activities can be easily found. The TFs with common activities can be explored for the pathogenesis of the disease. For investigating the deterioration mechanism or differences between subtypes and grades, the TFs with different activities should be further studied. The results of two additional experiments (one is the comparison of BC with no metastasis with BC in 3 grades, and other is the comparison of AD datasets in HIP with AD in 3 grades) and the biological analysis can be seen in the discussion part.

### Datasets

In this study, several publicly available microarray datasets of AD and BC are used to perform FastICA and NCA algorithms and the comparison analysis. They are series GSE5281, GSE1297 for AD [31, 32] and GSE42568 for BC [33] from NCBI PubMed (National Center for Biotechnology Information, U.S. National Library of Medicine, https://www.ncbi.nlm.nih.gov/pubmed). For GSE5281 dataset, it contains 161 samples from 6 areas of the human brain, including the entorhinal cortex (EC), hippocampus (HIP), medial temporal gyrus (MTG), posterior cingulate (PC), superior frontal gyrus (SFG) and primary visual cortex (VCX). Each sample contains 54675 gene probes for expression data by an Affymetrix Human Genome U133 Plus 2.0 Array (HG-U133_Plus_2). Each area contains the following two types of samples: samples from normal aging marked as controls and AD condition samples tagged as affected. In our study, here we used HIP expression data, which contains 13 control samples and 10 affected samples. For series GSE1297, it includes hippocampal gene expression of 9 control and 7 incipient, 8 moderate and 7 severe AD subjects. For GSE42568 dataset, it includes 104 breast cancer biopsies from patients aged between 31 years and 89 years at the time of diagnosis and 17 normal breast tissues. For the 104 BC samples, there are 45 BC patients with no metastasis and 59 BC patients with axillary lymph node metastasis samples. The Affymetrix Human Genome U133 Plus 2.0 Array (HG-U133_Plus_2) was used to measure the gene expression data with 54675 gene probes. In this study, the 17 control and 45 BC samples with no metastasis were used to perform the two-stage method.

## Results

### Feature gene extraction by FastICA

To perform FastICA on AD gene expression dataset, we used the data of hippocampus in GSE5281. the t-test was applied to 23 samples to reduce gene noise firstly, which displayed no distinct differences between the control and AD data. Then 8000 genes with t≧2.85 and p≦0.016446 were selected as the FastICA input. In FastICA algorithm, the nonlinear function *g*(*y*) = *tanh*(*a*1*y), where *a*1 is a constant, was used as the probability density distribution of the outputs *y* during the iterations. Considering the random initializations for the maximization of the constraint function and the problem of convergence to local optima by FastICA, we iterated FastICA 30 times to alleviate the instability of the slightly different results in each interaction. For each FastICA decomposition result, the extracted significant genes were different according to columns of ***A***, which matched the classification information in the rows in the gene expression matrix ***E***. We selected thousands of top genes as significant genes by calculating the number of significant genes. Finally, 2027 significant genes whose frequencies were greater than or equal to 21 were extracted as significant AD genes.

The same feature extraction process by FastICA was performed for the 17 control and 45 BC patient samples in the GSE42568 dataset. 1830 significant genes whose frequencies were greater than or equal to 20 were extracted as BC significant genes. Comparison of the 2027 AD significant genes with the 1830 BC significant genes showed that 267 shared genes were significantly differentially expressed in both AD and BC.

### NCA results and transcriptional regulatory process identification

The second stage in this study was to find the activities of the shared TFs and their regulatory influence on TGs by NCA. Two inputs are needed to perform NCA. One is a TG gene expression profiles matrix [***E***] and the other is an initial matrix [***C***_0_], a pre-defined regulatory influence matrix that provides the initial estimates of the influence of each TF on its TGs. To define key TFs and the original biologically qualitative regulatory influence on the TGs, the ITFP (integrated platform of mammalian transcription factors, http://connection.ebscohost.com/c/articles/34651881/itfp-integrated-platform-mammalian-transcription-factors) was used to identify significant TFs from the shared 267 genes. 40 TFs with 1212 TGs were identified. Through KEGG pathway analysis, 26 of the 40 TFs and their 237 TGs were identified in 37 biological pathways. Based on the KEGG analysis, we performed the NCA method on these 26 selected significant TFs with their 237 TGs for AD and BC.

**Fig 2:**
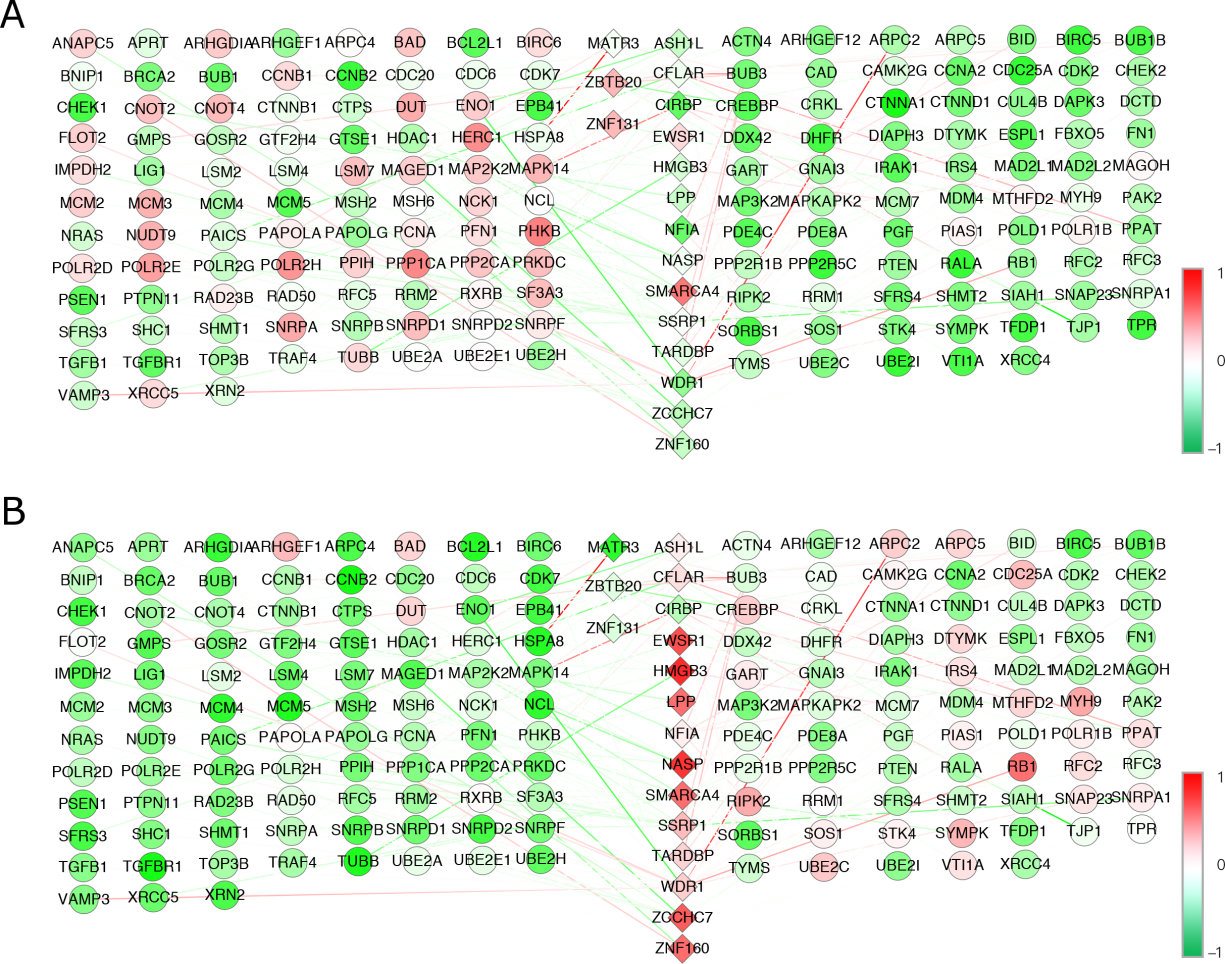
Dynamic transcriptional regulatory networks for the AD dataset. (A) presents the transcriptional regulatory network for the control samples, and (B) shows the transcriptional regulatory network for AD samples. The diamonds in the middle denote TFs with different colors according to their activity values. The different colored circles display TG gene expression, and the different colored lines between the TFs and TGs show the control strength.

For AD, the gene expression matrix [***E***] was a 237×23 matrix that denoted the microarray expression profiles of 237 TGs in 23 AD samples, including 13 control and 10 AD-affected samples. Matrix [***C***_0_] was a 237×26 matrix that represented the predefined connectivity strength of 237 TGs by 26 TFs. Element *c*_*ij*_ in matrix [***C***_0_] was set to 0 if there was no connectivity strength for TG *i* by TF *j*; otherwise, it was set to 1. To meet the uniqueness criteria requirements, matrix [***C***_0_] was cut down to a 166×17 matrix when performing NCA. Then, the final 17 TF activities on 23 samples (matrix [***P***]) and control strengths of the 17 TFs on 166 TGs (matrix [***C***]) were quantitatively obtained. Fig 2 shows the dynamic transcriptional regulatory networks from the 17 TFs on 166 TGs for the control (Fig 2 (A)) and AD-affected samples (Fig 2 (B)).

In Fig 2, 17 TFs are denoted by diamonds, with their activities values in different colors in the middle of the regulatory networks, and the gene expression of the 166 TGs are shown with circles on either side of the TFs. In these two sub-figures, TFs and TGs were placed in the same positions; this helps to easily detect dynamic changes between the control and AD-affected datasets. It is clear that the regulatory activity of the ASH1L, CFLAR, CIRBP, EWSR1, HMGB3, LPP, NASP, NFIA, SMARCA4, SSRP1, TARDBP, WDR1, ZCCHC7 and ZNF160 TFs increased from the control to the AD-affected samples. By contrast, the regulatory activities of the MATR3, ZBTB20 and ZNF131 TFs declined distinctly. Table 1 shows the KEGG pathway analysis of the selected 17 TFs and 166 TGs and shows the signaling pathways that are most relevant to these significant TFs and TGs.

**Table 1:** KEGG pathway analysis of the shared TGs in AD and BC.

Pathways	Number of genes
Pathways in cancer	39
Cell cycle	34
Spliceosome	29
Regulation of actin cytoskeleton	24
Neurotrophin signaling pathway	23
Ubiquitin mediated proteolysis	23
Purine metabolism	23
Pyrimidine metabolism	20
Oocyte meiosis	19
Tight junction	18
Chronic myeloid leukemia	17
Insulin signaling pathway	17
RNA degradation	16
Adherens junction	15
DNA replication	14
Progesterone-mediated oocyte maturation	14
Prostate cancer	14
Pathogenic Escherichia coli infection	13
ErbB signaling pathway	13
p53 signaling pathway	12
Renal cell carcinoma	12
Pancreatic cancer	12
Non-small cell lung cancer	12
Small cell lung cancer	12
TGF-beta signaling pathway	12
Gap junction	12
Endometrial cancer	11
Glioma	11
Nucleotide excision repair	10
Mismatch repair	8
Bladder cancer	8
One carbon pool by folate	7
SNARE interactions in vesicular transport	7
RNA polymerase	6
Homologous recombination	6
Thyroid cancer	6
Non-homologous end-joining	5

**Fig 3:**
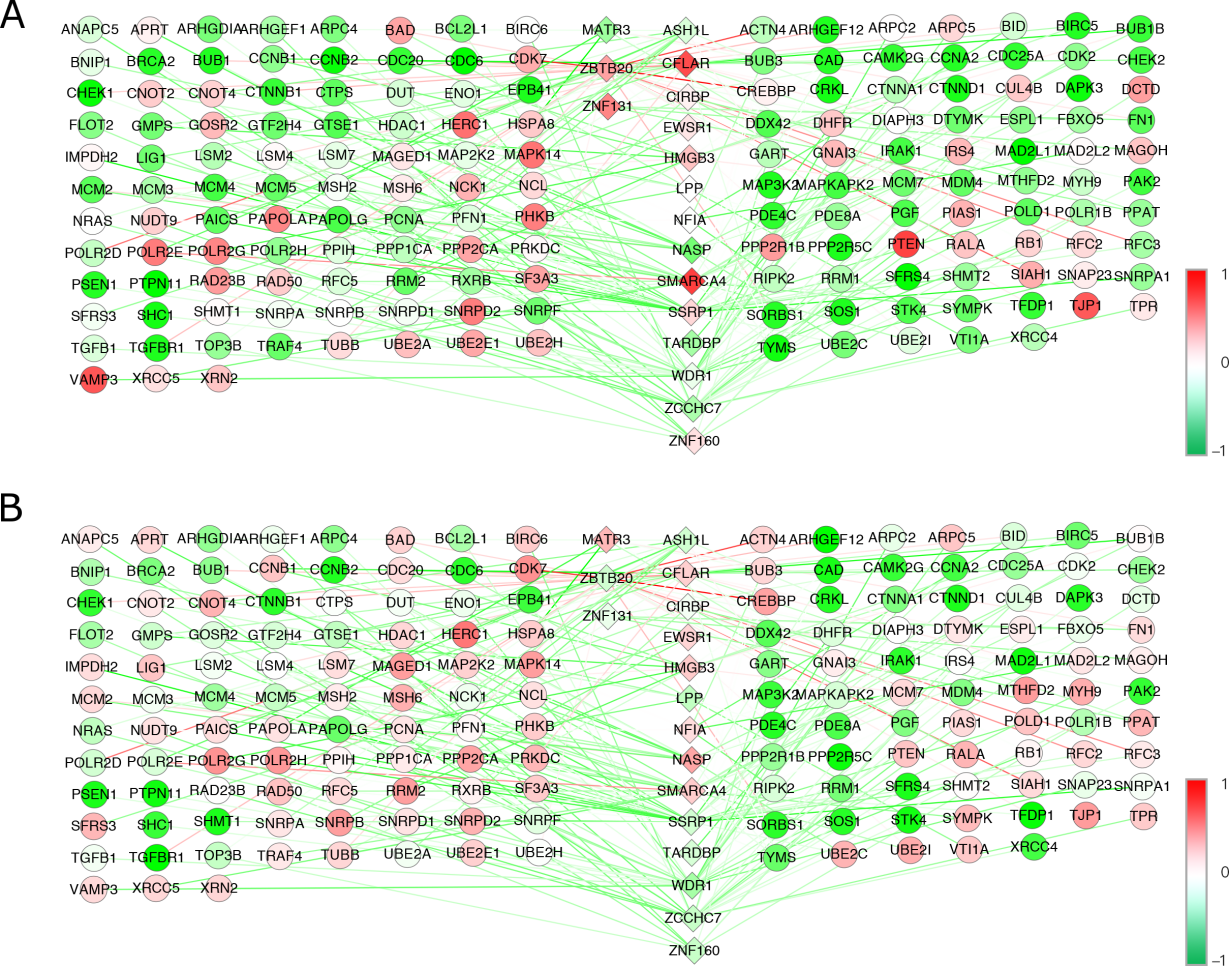
Dynamic transcriptional regulatory networks for the BC dataset. (A) presents the transcriptional regulatory network for the control samples, and (B) shows transcriptional regulatory network for the BC samples. The diamonds in the middle denote TFs, with different colors according to their activity values. The different colored circles display TG gene expression, and the different colored lines between the TFs and TGs show the control strength.

The NCA method was also applied to the BC transcriptional gene expression data. The selected 17 TF activities from 62 BC samples (matrix [***P***]) and the control strengths of the 17 TFs on the same 166 TGs (matrix [***C***]) were obtained after performing NCA. Similarly, Fig 3 shows the dynamic transcriptional regulatory networks for the 17 TFs on 166 TGs for the BC results.

The positions of the TFs and TGs in Fig 3 are the same as they were in Fig 2. According to these two sub-figures in Fig 3, we can clearly see that the regulatory activities of the EWSR1, MATR3, NASP, NFIA, TARDBP and ZCCHC7 TFs increased from the control to BC samples, while the activities of the ASH1L, CFLAR, CIRBP, HMGB3, LPP, SMARCA4, SSRP1, WDR1, ZBTB20, ZNF131 and ZNF160 TFs declined from the control to BC samples. Table 2 shows 17 TFs with their TGs and the increased or declined states (in up or down arrows) in AD and BC corresponding to their control samples.

**Table 2:** Common TFs with their TGs in AD and BC.

TFs	↑/↓	Description	TGs
ASH1L	AD**↑**BC**↓**	ash1 (absent, small, or homeotic)-like (Drosophila)	ARHGDIA, CNOT4, CRKL, ENO1, GART, HERC1, NCK1, PAPOLG, PFN1, POLR2E, PPP1CA, SNRPA
CFLAR	AD**↑**BC**↓**	CASP8 and FADD-like apoptosis regulator	BIRC5, BUB1B, BUB3, CAD, CCNA2, CCNB1, CDC20, CDC6, CHEK2, DTYMK, GMPS, LIG1, LSM4, MAD2L1, MCM4, MCM7, PAICS, PPAT, PRKDC, RFC5, TYMS
CIRBP	AD**↑**BC**↓**	cold inducible RNA binding protein	IRAK1, MAPK14, MTHFD2, RALA, UBE2A
HMGB3	AD**↑**BC**↓**	high-mobility group box 3	BIRC5, BUB1B, CAD, CCNB1, CDC20, CDC25A, GMPS, MCM3, MCM7, POLR2G, RRM2, TUBB, TYMS
LPP	AD**↑**BC**↓**	LIM domain containing preferred translocation partner in lipoma	BAD, BNIP1, CNOT2, PDE4C, PHKB, VTI1A
SMARCA4	AD**↑**BC**↓**	SWI/SNF related, matrix associated, actin dependent regulator of chromatin, subfamily a, member 4	CAD, CDK2, DTYMK, ESPL1, GTF2H4, LSM4, MAD2L1, MAP2K2, MCM2, MCM3, MCM4, MCM7, MSH6, POLR2E, PRKDC, RFC5, SNRPA, SYMPK, TFDP1, TRAF4, TUBB, TYMS, UBE2I, XRCC5
SSRP1	AD**↑**BC**↓**	structure specific recognition protein 1	APRT, BUB1, BUB3, CAD, CCNA2, CDC25A, CHEK1, CTNND1, CTPS, DHFR, DIAPH3, GART, GTSE1, IMPDH2, LIG1, LSM2, LSM4, LSM7, MAD2L1, MCM3, MCM4, MCM7, MSH6, PAICS, POLD1, POLR2G, POLR2H, PPIH, PRKDC, RFC2, RFC3, RFC5, RRM1, SF3A3, SHMT1, SHMT2, SNRPA, SNRPA1, SNRPB, SNRPD1, SNRPD2, SNRPF, TYMS, UBE2C
WDR1	AD**↑**BC**↓**	WD repeat domain 1	ARHGDIA, ARPC2, ARPC4, ARPC5, BCL2L1, CTNNA1, DAPK3, DCTD, ENO1, FLOT2, GNAI3, GOSR2, MAPKAPK2, MYH9, NRAS, PDE8A, POLR2E, PSEN1, PTPN11, RB1, RXRB, SHC1, SNAP23, TGFB1, VAMP3, XRN2
ZNF160	AD**↑**BC**↓**	zinc finger protein 160	ARHGEF1, CNOT2, IRS4, PDE4C PGF, POLR1B, PPP2CA, RAD50, TOP3B
MATR3	AD**↑**BC**↑**	martin 3	BUB3, CUL4B, DDX42, FN1, HSPA8, PAPOLA, RB1
EWSR1	AD**↑**BC**↑**	Ewing sarcoma breakpoint region 1	SFRS3, SNRPA, SNRPB
NASP	AD**↑**BC**↑**	nuclear auto antigenic sperm protein (histone-binding)	BUB1B, CCNB2, DUT, FBXO5, GTSE1, HDAC1, MCM2, MCM3, MCM5, MCM7, MSH2, NCL, PCNA, TYMS, UBE2C
NFIA	AD**↑**BC**↑**	Nuclear factor I/A	BID, BUB3, ESPL1, MAD2L2, MAPK14, NRAS, RIPK2
TARDBP	AD**↑**BC**↑**	TAR DNA binding protein	BUB3, PPP2R1B
ZCCHC7	AD**↑**BC**↑**	zinc finger, CCHC domain containing 7	ANAPC5, BIRC6, BRCA2, CUL4B, EPB41, GART, MAGED1, MAP3K2, MDM4, NUDT9, PAK2, PIAS1, PPP2R5C, PTEN, RAD23B, SFRS4, SOS1, STK4, TPR, UBE2E1, UBE2H, XRCC4
ZBTB20	AD**↓**BC**↓**	zinc finger and BTB domain containing 20	ACTN4, ARHGEF12, BAD, BID, BUB1, BUB3, CDC20, CDC6, CDK7, CHEK1, CREBBP, CTNNB1, GART, GNAI3, GTSE1, IRAK1, MAD2L1, MCM5, NRAS, PAK2, PDE4C, POLR2D, RAD50, RALA, RFC2, RFC3, SIAH1, SORBS1, TFDP1, TGFBR1, TJP1, TOP3B, TUBB, UBE2H, UBE2I
ZNF131	AD**↓**BC**↓**	Zinc finger protein 131	CAMK2G, FBXO5, FN1, MAGOH, PPAT

## Discussion

### Comparison of several feature gene extraction methods

In recent years, several matrix decomposition methods have been widely used for feature gene extraction, gene clustering and disease classification, including principle component analysis (PCA) or singular value decomposition (SVD), ICA and nonnegative matrix factorization (NMF). PCA or SVD is a statistical technique with the advantage of dimensionality reduction under the assumption that decomposition of the data possesses an orthogonal structure. ICA is also an unsupervised learning method that is used to identify biologically significant processes by statistically independent basic functions. NMF decomposes high-dimensional gene expression data into positive metagenes with a local biological representation based on non-negative constraints, which is more natural than representing gene expression profiles. To compare the feature gene extraction differences of these methods, we performed PCA, ICA and NMF on AD gene expression data in our previous studies [34–36].

From the PCA results, the identified significant AD genes showed that they are mainly related to immunoreactions, metal proteins, membrane proteins, lipoproteins, neuropeptides, cytoskeleton proteins, binding proteins, ribosomal proteins and phosphoric proteins [34]. The limitation of the PCA model is that the number of significant genes is much fewer than ICA and NMF.

By performing NMF, more than 1500 significant AD genes were identified. A large number of these genes were related to metal metabolism and inflammation, cell growth, cell cycle, apoptosis, cellular fission and cell repair [35]. The shortcomings of the NMF method were that the number of significant genes was hard to reduce and that the local characteristics that appeared among the metagenes were not clear in our data.

From the biological analysis of the FastICA results on the same dataset, we found that the significant genes were involved in immunoreactions, metal proteins, membrane proteins, lipoproteins, neuropeptides, cytoskeleton proteins, binding proteins and ribosomal proteins and play prominent roles in AD phenotypes. FastICA also found many oncogenes and phosphoric proteins that were significantly lowly expressed in AD.

Based on the molecular biological analysis of these three types of results, significant gene extraction by ICA was better than those by PCA and NMF at identifying known and novel genes in meaningful biological processes for AD. The comparison indicated that ICA is more efficient at extracting potentially relevant genes from microarray data as well as mapping data that is closer to AD pathogenesis. Moreover, all three of these methods are based on purely statistical constraints and do not use any biological knowledge or transcriptional regulatory information. Therefore, their results cannot contain biological transcriptional regulatory networks, which is also a primary reason that we used network component analysis (NCA) to detect the disease transcriptional regulatory mechanisms after feature gene extraction.

### Comparison of different datasets with the same methods

In this study, besides the above main experiments of discovering the inverse TFs activities between AD and BC, two other experiments for exploring the regulatory mechanism for different subtypes or grades for the same disease (AD or BC) are studied as well. One is the experiment of AD in HIP with AD in 3 varying severities, the other is the comparison of BC with no metastasis with BC in 3 grades.

i) AD data in hippocampus vs. AD data in varying severities

**Fig 4:**
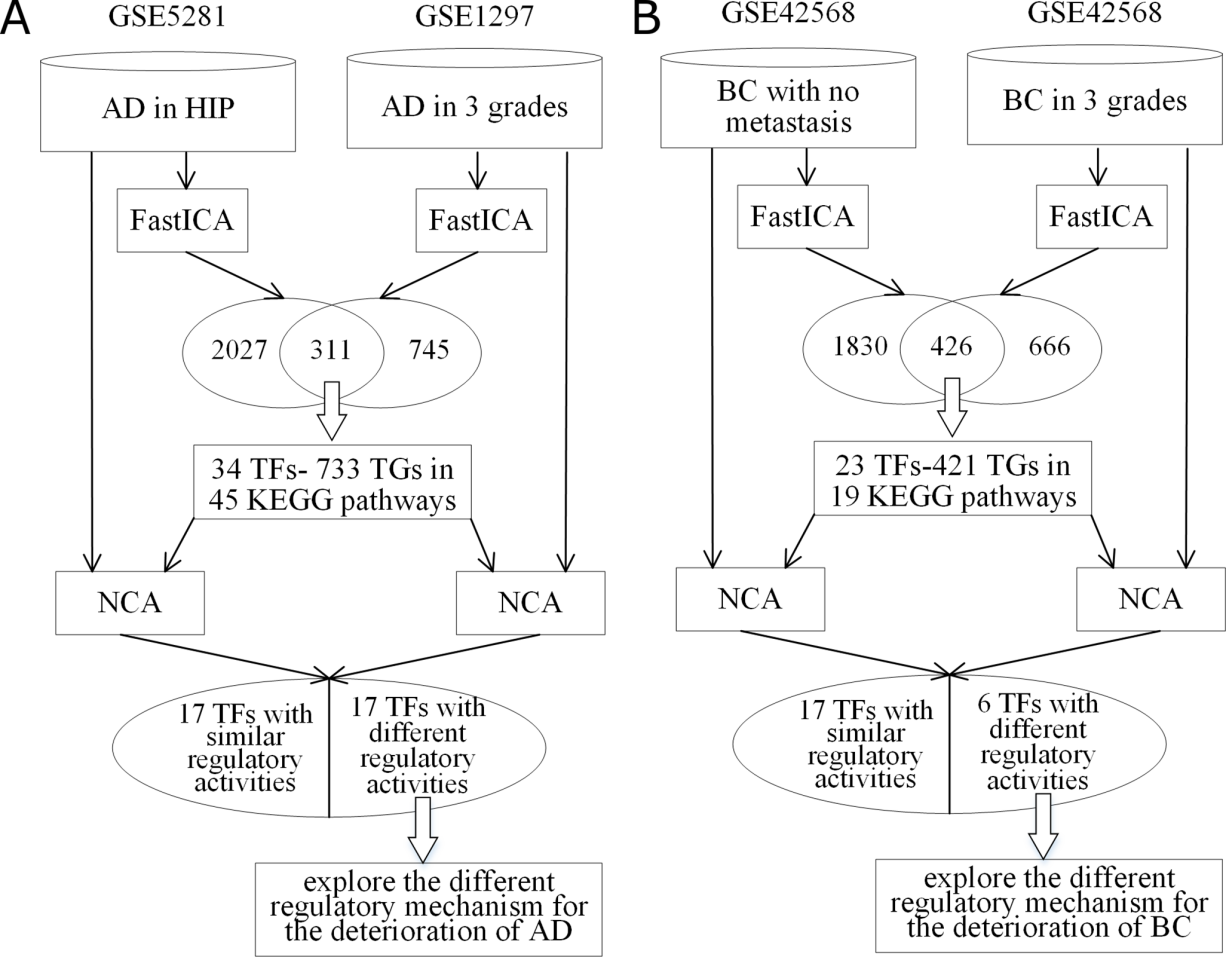
Flowchart of the experiments for different datasets of one disease (AD/BC). (A) gives the flowchart of the two-stage procedure in comparing AD-HIP dataset with AD in 3 severities dataset; (B) gives the flowchart of the two-stage procedure in comparing BC with no metastasis dataset with BC in 3 grades dataset.

To explore the differently regulatory mechanism between varying severities of AD, another AD dataset, series GSE1297 [37], was studied as well. GSE1297 dataset includes hippocampal gene expression of 9 control and 7 incipient, 8 moderate and 7 severe AD subjects. FastICA was performed to these three varying severities data and 245, 268 and 324 significant genes were extracted respectively from the incipient, moderate and severe AD samples [32]. To compare this dataset with the above results of AD dataset GSE5281, the shared genes between these two AD datasets were extracted. There are 311 shared genes between these two datasets and 50 out of them are also shared with the 267 significant genes. 34 TFs with 733 TGs in 45 KEGG pathways were identified (the flowchart can be seen in Fig 4 (A)). Then, NCA algorithm was performed to explore the similarities and differences of the activities of the 34 TFs between these two datasets. For the first 17 TFs, their regulatory activities are similar, while the regulatory activities of other 17 TFs are in opposite directions. Table 3 shows the upregulated or downregulated TF activities in these two datasets by the up or down arrows.

**Table 3:** Regulatory activities comparison of the shared TFs in two different AD datasets.

TF	Description	AD-HIP	AD in 3 severities
CEBPD	CCAAT/enhancer binding protein (C/EBP), delta	**↑**	**↑**
CLTC	clathrin, heavy chain (Hc)	**↑**	**↑**
DDX3X	DEAD (Asp-Glu-Ala-Asp) box polypeptide 3, X-linked	**↑**	**↑**
GNB1	guanine nucleotide binding protein (G protein), beta polypeptide 1	**↑**	**↑**
JUND	jun D proto-oncogene	**↑**	**↑**
KLC1	kinesin light chain 1	**↑**	**↑**
LANCL1	LanC lantibiotic synthetase component C-like 1 (bacterial)	**↑**	**↑**
NFIB	nuclear factor I/B	**↑**	**↑**
PAFAH1B1	platelet-activating factor acetylhydrolase 1b, regulatory subunit 1 (45kDa)	**↑**	**↑**
PEX5	peroxisomal biogenesis factor 5	**↑**	**↑**
ZBTB20	zinc finger and BTB domain containing 20	**↑**	**↑**
FHL1	four and a half LIM domains 1	**↓**	**↓**
NCL	nucleolin	**↓**	**↓**
PEA15	phosphoprotein enriched in astrocytes 15	**↓**	**↓**
QKI	quaking homolog, KH domain RNA binding (mouse)	**↓**	**↓**
R3HDM1	R3H domain containing 1	**↓**	**↓**
ZNF160	zinc finger protein 160	**↓**	**↓**
DDX17	DEAD (Asp-Glu-Ala-Asp) box polypeptide 17	**↑**	**↓**
DICER1	dicer 1, ribonuclease type III	**↑**	**↓**
GLS	glutaminase	**↑**	**↓**
GTF3A	general transcription factor IIIA	**↑**	**↓**
HMGB1	high-mobility group box 1	**↑**	**↓**
TTC3	tetratricopeptide repeat domain 3	**↑**	**↓**
ANK2	ankyrin 2, neuronal	**↓**	**↑**
CIRBP	cold inducible RNA binding protein	**↓**	**↑**
JUN	jun D proto-oncogene	**↓**	**↑**
NFE2L1	nuclear factor (erythroid-derived 2)-like 1	**↓**	**↑**
NFKBIA	nuclear factor of kappa light polypeptide gene enhancer in B-cells inhibitor, alpha	**↓**	**↑**
RBM8A	RNA binding motif protein 8A	**↓**	**↑**
RPS4Y1	ribosomal protein S4, Y-linked 1	**↓**	**↑**
RPS11	ribosomal protein S11	**↓**	**↑**
SPTBN1	spectrin, beta, non-erythrocytic 1	**↓**	**↑**
TNPO1	transportin 1	**↓**	**↑**
TRIM2	tripartite motif-containing 2	**↓**	**↑**

Table 3 shows that the regulatory activities of the first 17 TFs: CEBPD, CLTC, DDX3X, GNB1, JUND, KLC1, LANCL1, NFIB, PAFAH1B1, PEX5, ZBTB20, FHL1, NCL, PEA15, QKI, R3HDM1 and ZNF160 are in the same directions. The molecular biological analysis showed that the changes of these TF activities and their target genes in the interactions of signaling proteins in cell cycle, chronic inflammation and immune response play important roles in the deterioration of AD.

By contrast, the regulatory activities of the other 17 TFs: DDX17, DICER1, GLS, GTF3A, HMGB1, TTC3, ANK2, CIRBP, JUN, NFE2L1, NFKBIA, RBM8A, RPS4Y1, RPS11, SPTBN1, TNPO1 and TRIM2 show great changes during the deterioration. The biological analyses of the regulatory activities show the transcriptional changes for the deterioration of AD. As we know that, in eukaryotic, the way to initiate transcription eukaryotic RNA polymerase requires the assistance of proteins. Among these 17 TFs, many of them are related to the regulation of RNA, such as DDX17, DICER1, CIRBP, RBM8A, RPS4Y1, RPS11 and TNPO1. It suggests that with the aggravation of AD, the regulation of RNA level changed greatly and they will lead to the changes of the expression of many proteins. Additionally, the results show that some of the TFs are associated with inflammation and they are incessantly upregulated during the deterioration of AD such as NFE2L1 and NFKBIA.

ii) BC data with no metastasis vs. BC in different grades

In this experiment, we compare the BC dataset with no metastasis with BC in 3 grades to explore the regulatory mechanism for the deterioration of BC. The two-stage procedure was performed on BC series GSE42568, which is the same dataset in current study but in a different classification rule. The 104 breast cancer samples were divided into 3 categories according to their histologic grading: 11 tumours were grade 1; 40 were grade 2 and 53 were grade 3. in the similar way, FastICA was performed to these three BC grades data respectively. 666 common significant genes for three grades were extracted [38]. To compare this experiment with the above results of BC data with no metastasis, the shared genes between these two BC datasets were further analysed. There are 426 shared genes between these two datasets and 47 out of them are also shared with the 267 significant genes of the first experiment. 23 TFs with 421 TGs which take part in the biological functions of 19 KEGG pathways were identified, and NCA algorithm was performed to explore the similarities and differences of the activities for the 23 TFs (Fig 4 (B) shows the flowchart). Table 4 displays the activities of the shared TFs in these two datasets by up or down arrows.

**Table 4:** The regulatory activities of the shared TFs in two different BC datasets.

TF	Description	BC with no metastasis	BC in 3 grades
ANK3	ankyrin 3, node of Ranvier (ankyrin G)	**↑**	**↑**
CYR61	cysteine-rich, angiogenic inducer, 61	**↑**	**↑**
ESR1	estrogen receptor 1	**↑**	**↑**
FOSB	FBJ murine osteosarcoma viral oncogene homolog B	**↑**	**↑**
HMGB3	high-mobility group box 3	**↑**	**↑**
IRX5	iroquois homeobox 5	**↑**	**↑**
MYB	v-myb myeloblastosis viral oncogene homolog (avian)	**↑**	**↑**
PRICKLE2	prickle homolog 2 (Drosophila)	**↑**	**↑**
RETSAT	retinol saturase (all-trans-retinol 13,14-reductase)	**↑**	**↑**
SPTBN1	Spectrin, beta, non-erythrocytic 1	**↑**	**↑**
ZBTB16	zinc finger and BTB domain containing 16	**↑**	**↑**
DOK7	docking protein 7	**↓**	**↓**
ECT2	epithelial cell transforming sequence 2 oncogene	**↓**	**↓**
FHL1	four and a half LIM domains 1	**↓**	**↓**
FOS	FBJ murine osteosarcoma viral oncogene homolog	**↓**	**↓**
KRT19	keratin 19	**↓**	**↓**
MESP1	mesoderm posterior 1 homolog	**↓**	**↓**
DMD	dystrophin	**↑**	**↓**
EPS8L2	EPS8-like 2	**↑**	**↓**
NKX3-1	NK3 homeobox 1	**↑**	**↓**
PPP2R1B	protein phosphatase 2, regulatory subunit A, beta	**↑**	**↓**
RNF150	ring finger protein 150	**↓**	**↑**
SPDEF	SAM pointed domain containing Ets transcription factor	**↓**	**↑**

From Table 4 we can see, for the first 17 TFs, their regulatory activities are in the same directions, such as ANK3, CYR61, ESR1, FOSB, HMGB3, IRX5, MYB, PRICKLE2, RETSAT, SPTBN1 and ZBTB16 are upregulated in both BC datasets, and DOK7, ECT2, FHL1, FOS, KRT19 and MESP1 are down-regulated in these two BC datasets. It is clear to find that the continuous activation or inhibition of these TFs are closely associated with the deterioration of breast cancer in the signal transduction pathways like cell proliferation, mitosis, apoptosis, Ras signal transduction, DNA replication, cholesterol homeostasis and growth regulation. While the regulatory activities of the other 6 TFs including DMD, EPS8L2, NKX3-1, PPP2R1B, RNF150 and SPDEF show opposite regulatory activities between these two BC datasets. The biological analyses of the inverse regulatory activities help us to exploring the transcriptional mechanism from BC with no metastasis to the deterioration of BC. For example, NKX3-1 is a tumor suppressor gene and PDEF is an oncogene. NKX3-1 can interact with cancer derived Ets factor (PDEF) and suppress the ability of PDEF to transactivate the cancer specific promoter. Our results show that during the deterioration of BC, NKX3-1 is downregulated continually while SPDEF is upregulated at the same time.

From the results of the above experiments, we can conclude that the quantification of the changes of significant TFs provide an increased understanding to the regulatory laws of disease in varying severity, different grades or even different diseases. The extracted significant genes and TFs on different datasets of one disease were not the same, since FastICA extracts the statistically independent biological process and feature genes based on the gene expression profiles of each dataset. However, it is interesting that the molecular biological analysis showed that the TFs related pathways were closely similar like mitosis, apoptosis, cell cycle, chronic inflammation and immune disorder and so on. That means the method can find out the key regulatory changes of one disease. To exploring the regulatory mechanism for different severities, grades, subtypes or different diseases, selecting the common significant TFs and reconstructing their quantitatively regulatory networks are effective to gain insights into the pathogenesis of diseases.

### Transcriptional regulatory processes related to immune response between AD and BC

There are many kinds of neurodegenerative diseases, among them AD is the largest proportion of the diseases, and younger trend. Parkinson disease (PD) is the second most common neurodegenerative disease. Among people over age 60, there is a 2–4% for risk of Parkinson [39,40]. Amyotrophic lateral sclerosis (ALS) and multiple sclerosis (MS) also are neurodegenerative diseases that affect the central nervous system, both attack the body’s nerves and muscles and be low incidence [41,42]. The causes of neurodegenerative diseases are not completely clear, but a lot of evidence have proved with the related inflammation and immune changed. In cancer studies, many results show the Immune response plays an important role in tumour development, treatment and prognosis. Tumours in different stages have different characteristics. However, inflammation and immune changes in tumour growth and also plays an important role in the process of treatment [43, 44]. In our research, the transcriptional regulatory pathway of inflammation and immune disorders of AD and BC were first studied, and other kinds of neurodegenerative diseases and cancers will be studied in the next step.

Transcription factors are a diverse family of proteins that generally function in multi-subunit protein complexes that are vital to the normal development of an organism and are involved in routine cellular functions and responses to disease. The function of TFs allows for the unique expression of each gene in different cell types and during development. Therefore, it is very important to study TFs while analyzing pathways to understand disease pathogenesis. From the TF activities from dynamic transcriptional regulatory networks in AD and BC (Figs 2 and 3), we found that they shared 17 TFs, as calculated by NCA, that play important roles in inflammation and the immune response in both AD and BC. Specifically, 10 out of 17 TFs showed inverse regulatory activities between AD and BC (top 10 TFs in Table 2), which showed that these two diseases shared many genes and biological pathways, but that the genes and pathways are regulated in different directions of the same spectrum. Combined with the current understanding of the functions of the innate and adaptive immune systems, transcriptional biological analyses related to the immune response were reviewed below to determine the opposite pathogenic regulatory mechanisms of AD and BC.

Ascl1 (ASH1L), the activity of which increased in AD and declined in BC compared to control samples in the NCA results, is central to the differentiation of neuroblasts and the lateral inhibition mechanism, which inherently creates a safety net in the event of damage or death in these incredibly important cells, as well as neuronal commitment [45]. Ascl1 regulates astrocytes and oligodendrocytes by density and distribution in neurodegenerative diseases [46,47]. Under certain conditions, ASH1L can regulate interleukin-6 production. As a cytokine, interleukin-6 production can regulate NF-κB, an important nuclear factor that is closely related to inflammation, by mitogen activated protein kinase (MAPK) pathways [48–50]. As a TF, if ASH1L displays abnormal regulation, it will lead to many diseases, such as cancer and neurological diseases [51–52]. ASH1L has been implicated in facioscapulohumeral muscular dystrophy. In this disease, human muscles will experience progressive wasting [53], which is a common feature of AD, i.e., cells progressively degenerate in both diseases, and ASH1L appears at the opposite end of BC.

The p53 gene is a tumor suppressor gene that is involved in anti-tumor formation and inducing cell apoptosis. CFLAR (C-FLIP) can suppress caspase 8 activation and mediate apoptosis [54]. However, some studies have reported that p53 can upregulate CFLAR, inhibit NF-κB-regulated gene transcription and induce cell death in a caspase-8-independent manner. In the NCA results, the CFLAR activities increased in AD and decreased in BC. Data from the literature show that CFLAR is also regulated by the IL-2 and MAPK pathway [55,56] and that abnormal expression is related to some diseases, such as cancer and autoimmune diseases [57].

Cold-inducible RNA binding (CIRB) protein (CIRBP) is a TF that plays a critical role in controlling cellular response upon confronting a variety of cellular stresses, including short wavelength ultraviolet light, neuroinflammation, hypothermia and hypoxia [58–61]. Some studies have indicated that CIRP regulates multiple pathways, such as MAPK, Wnt, apoptosis and many cancer-related signaling pathways in cerebral ischemia [62,63]. It has also been reported to mediate neuroinflammation [64]. From our transcriptional regulatory network figures, we identified the inverse regulation of CIRBP between AD and BC, which increased in AD and declined in BC.

Hmgb3 is a member of high mobility group DNA-binding motifs, which have been found to increase the transcriptional regulatory process in AD and decrease this process in BC. It is known that Hmgb3 over-expression can inhibit B-cell and myeloid differentiation. Therefore, reducing the regulation of the HMGB3 protein levels is an important step for myeloid and B-cell differentiation [65]. HMGBs bind to all of the immunogenic nucleic acids examined with a correlation between affinity and immunogenic potential. Its suppression by small interfering RNA led to impaired activation of the Irf3 and NF-κB transcription factors [66]. HMGB expression disorders can lead to immunological disorders and some diseases, such as cancer, neurological diseases, and microphthalmia syndromic [67].

In the transcriptional regulatory network figures from our research, lipoma preferred partner (LPP) was shown to increase in AD and decline in BC. LPP plays a structural role at sites of cell adhesion in maintaining cell shape and motility. In addition to these structural functions, it is also implicated in signaling events and gene transcription activation. The LPP protein is localized at sites of cell adhesion, such as focal adhesions and cell-cell contacts, and shuttles to the nucleus where it has transcriptional activation capacities [67–69]. LPP and the expression of fusion proteins probably mediate tumor growth [70]. Some studies have reported that LPP correlated innate and T cell-mediated immune responses [71]. This may be additional evidence that AD and BC are both closely associated with the immune response, but at opposite ends.

The SMARCA4 (BRG1, SWI/SNF) protein belongs to the SWI/SNF family. Their members have helicase and ATPase activities and are thought to regulate the transcription of certain genes by altering the chromatin structure around those genes. A previous report has shown that SWI/SNF regulates T cell inactivation and growth via cytokine promotion. Therefore, SWI/SNF plays an important role in subsequent immune responses [72]. SWI/SNF expression disorders have been found to lead to cancer and AD [73,74]. In the NCA results, we observe that SMARCA4 is increased in AD and decreased in BC.

In the transcriptional regulatory networks, SSRP1 was shown to increase in AD and decrease in BC. SSRP1 (FACT) is component of the FACT complex, a general chromatin factor that acts to reorganize nucleosomes. The FACT complex is involved in multiple processes that require DNA as a template, including mRNA elongation, DNA replication and DNA repair. During transcription elongation, the FACT complex acts as a histone chaperone that both destabilizes and restores the nucleosomal structure. FACT and cisplatin-damaged DNA may be crucial to the anticancer mechanism of cisplatin [75].

WD-repeat protein 1 (WDR1) or actin-interacting protein 1 (AIP1) is a highly conserved WD-repeat protein in eukaryotes that promotes cofilin-mediated actin filament disassembly [76]. The transcriptional regulatory results show that WDR1 was inversely regulated between AD and BC. It is an emerging regulator of the actin cytoskeleton that is vital to filament disassembly. When WDR1 loses its function, it leads to embryonic lethality, macrothrombocytopenia and autoinflammatory disease [77].

ZNF160 is shown to increase in AD and decline in BC. This TF represses the tlr4 gene, a protein encoded by the Toll-like receptor (TLR) family, which plays a fundamental role in pathogen recognition and activation of innate immunity by cytokines [78]. Under certain conditions, TLR4 can increase AML-reactive T cell generation and suppress oncoproteins from human papillomavirus E7 and E6 proteins [79].

MATR3 is a protein binding RNA and DNA. It may play a role in transcription or interact with other nuclear matrix proteins to form the internal fibrogranular network. It interacts with TDP-43 to cause amyotrophic lateral sclerosis (ALS) and frontotemporal dementia [80]. A proteomic screen revealed that MATR3 is bound to calmodulin and suggested that it is cleaved by both caspase-3 and caspase-8 [81,82]. In the transcriptional regulatory process, the activities of this TF were increased in AD and declined in BC.

**Fig 5:**
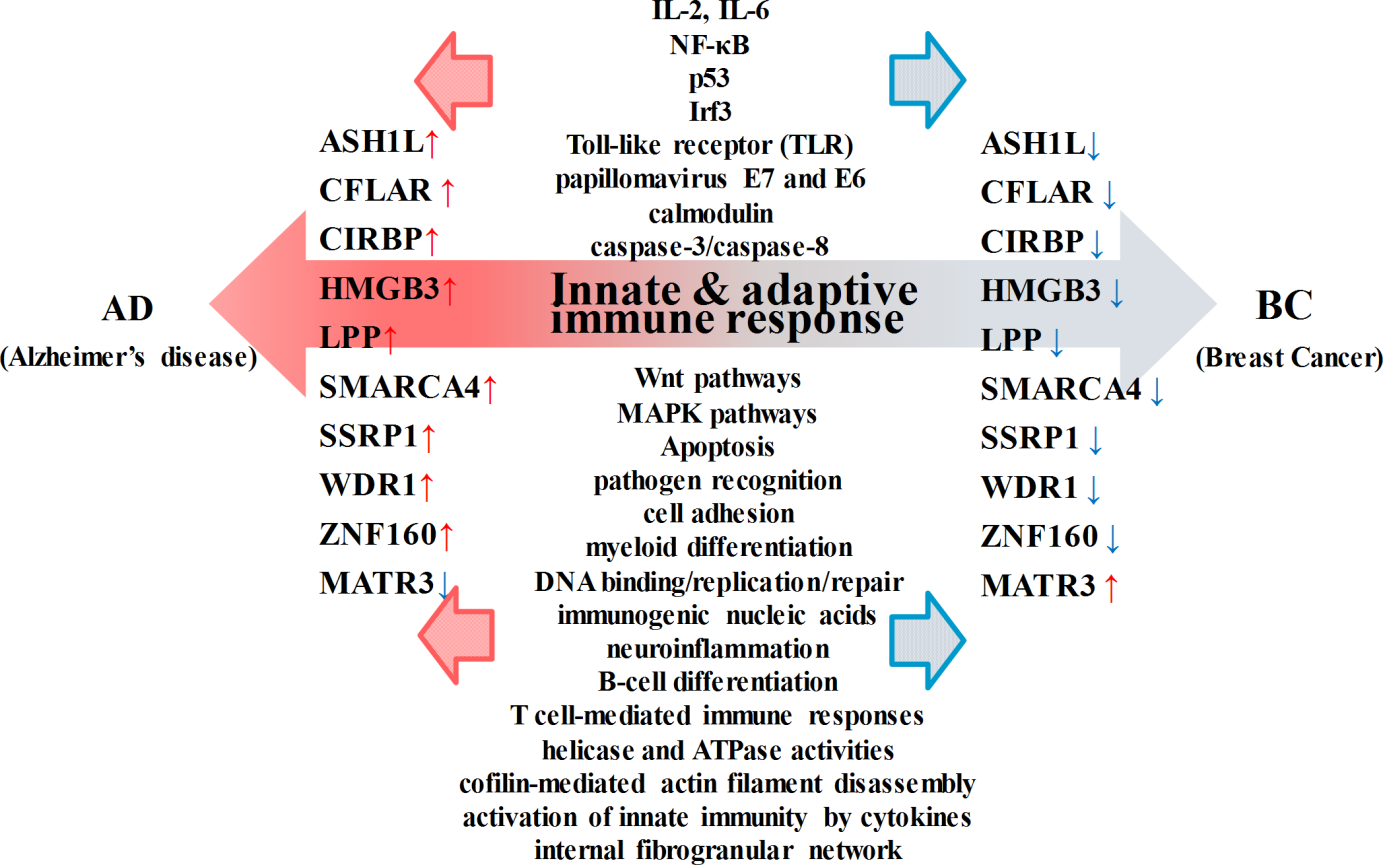
The inversely associated TFs in Table 2 and their related genes, pathways and biological processes. This figure shows the inverse regulatory activities of the common TFs and pathways between AD and BC. From the biological analysis we can know that they are closely related to innate and adaptive immune response.

Fig 5 displays the related target genes, pathways and common pathophysiological mechanisms with the opposing ends of the 10 inversely regulated TFs in the immune response between AD and BC.

In Fig 5, the common TFs between AD and BC with inverse regulatory activities are displayed on both sides of the horizontal axis towards these two diseases. The genes, pathways and biological processes regulated by or related to the common TFs are arranged on the vertical axis and are involved in the MAPK pathway, Wnt pathway, inflammation, apoptosis, DNA binding/replication/repair, T cell mediated immune response, and so on, which are closely associated with innate and adaptive immune responses and play important roles in the pathogenesis of both AD and BC.

To investigate how the activities of these 17 TFs on the 166 TGs change in the deterioration of these two diseases, we performed the NCA algorithm of these TFs and TGs on the additional AD and BC datasets respectively. The results are showed in Tables 5 and 6.

**Table 5:** The regulatory activities of the 17 TFs in the additional AD datasets.

TFs	Description	AD incipient	AD moderate	AD severe
SSRP1	structure specific recognition protein 1	**↑**	**↑**	**↑**
ZNF160	zinc finger protein 160	**↑**	**↑**	**↑**
ASH1L	ash1 (absent, small, or homeotic)-like (Drosophila)	**↑**	**↑**	**↓**
NFIA	Nuclear factor I/A	**↑**	**↑**	**↓**
ZBTB20	zinc finger and BTB domain containing 20	**↑**	**↑**	**↓**
ZCCHC7	zinc finger, CCHC domain containing 7	**↑**	**↑**	**↓**
CFLAR	CASP8 and FADD-like apoptosis regulator	**↑**	**↓**	**↑**
TARDBP	TAR DNA binding protein	**↑**	**↓**	**↓**
EWSR1	Ewing sarcoma breakpoint region 1	**↓**	**↑**	**↑**
NASP	nuclear auto antigenic sperm protein (histone-binding)	**↓**	**↑**	**↑**
CIRBP	cold inducible RNA binding protein	**↓**	**↓**	**↑**
MATR3	martin 3	**↓**	**↓**	**↑**
ZNF131	Zinc finger protein 131	**↓**	**↓**	**↑**
HMGB3	high-mobility group box 3	**↓**	**↓**	**↓**
LPP	LIM domain containing preferred translocation partner in lipoma	**↓**	**↓**	**↓**
SMARCA4	SWI/SNF related, matrix associated, actin dependent regulator of chromatin, subfamily a, member 4	**↓**	**↓**	**↓**
WDR1	WD repeat domain 1	**↓**	**↓**	**↓**

**Table 6:** The regulatory activities of the 17 TFs in the additional BC datasets.

TFs	Description	BC gradeⅠ	BC gradeⅡ	BC grade Ⅲ
CIRBP	cold inducible RNA binding protein	**↑**	**↑**	**↓**
TARDBP	TAR DNA binding protein	**↑**	**↑**	**↓**
NASP	nuclear auto antigenic sperm protein (histone-binding)	**↑**	**↓**	**↑**
NFIA	Nuclear factor I/A	**↑**	**↓**	**↑**
HMGB3	high-mobility group box 3	**↑**	**↓**	**↑**
ZCCHC7	zinc finger, CCHC domain containing 7	**↑**	**↓**	**↑**
SSRP1	structure specific recognition protein 1	**↑**	**↓**	**↓**
WDR1	WD repeat domain 1	**↑**	**↓**	**↓**
ZNF131	Zinc finger protein 131	**↑**	**↓**	**↓**
MATR3	martin 3	**↓**	**↑**	**↑**
ASH1L	ash1 (absent, small, or homeotic)-like (Drosophila)	**↓**	**↑**	**↓**
EWSR1	Ewing sarcoma breakpoint region 1	**↓**	**↑**	**↓**
LPP	LIM domain containing preferred translocation partner in lipoma	**↓**	**↑**	**↓**
ZBTB20	zinc finger and BTB domain containing 20	**↓**	**↓**	**↑**
CFLAR	CASP8 and FADD-like apoptosis regulator	**↓**	**↓**	**↑**
SMARCA4	SWI/SNF related, matrix associated, actin dependent regulator of chromatin, subfamily a, member 4	**↓**	**↓**	**↓**
ZNF160	zinc finger protein 160	**↓**	**↓**	**↓**

Table 5 shows the changes of activities of the 17 TFs during varying severities of AD. It is clear to find that SSRP1 and ZNF160 are continuously activated during the deterioration of AD; ASH1L, NFIA, ZBTB20, ZCCHC7 are upregulated in the incipient and moderate AD but downregulated in the severe AD; CFLAR and TARDBP are upregulated at the beginning of AD and downregulated during the deterioration; by contrast, EWSR1, NASP, CIRBP, MATR3 and ZNF131 are downregulated at the beginning of AD and upregulated when the disease gets worse; and the activities of HMGB3, LPP, SMARCA4 and WDR1 continuously decrease with the deterioration of AD.

Table 6 provides the activities of the 17 TFs in 3 grades of BC dataset. The changes of the activities of these 17 TFs are more complicated than those in AD. CIRBP, TARDBP, NASP, NFIA, HMGB3, ZCCHC7, SSRP1, WDR1, ZNF131 and MATR3 are upregulated in the grade Ⅰof BC, but downregulated in either grade Ⅱor grade Ⅲ; among them, some TFs like NASP, NFIA, HMGB3 and ZCCHC7 are even upregulated again in grade Ⅲ; by contrast, MATR3, ASH1L, EWSR1, LPP, ZBTB20, CFLAR are downregulated at the beginning of BC but upregulated in grade Ⅱor grade Ⅲ, sometimes they downregulated again when the disease getting into terminal period; SMARCA4 and ZNF160 continuously decrease with the deterioration of BC.

It can be seen that the regulatory status of these 17 TFs in different course of AD and BC are much different. In order to explore the regulatory status of the 10 inversely associated TFs discussed in the AD-BC experiment in the similar course of diseases, we extract the TFs with opposite association and compare their activities of incipient AD with BC in grade Ⅰ, the moderate AD with BC in grade Ⅱ and severe AD with BC in grade Ⅲ respectively (see Tables 7, 8 and 9).

**Table 7:** The TFs with inverse activities of incipient AD and BC in grade Ⅰ.

TFs	Description	AD incipient	BC gradeⅠ
ASH1L	ash1 (absent, small, or homeotic)-like (Drosophila)	**↑**	**↓**
CFLAR	CASP8 and FADD-like apoptosis regulator	**↑**	**↓**
ZNF160	zinc finger protein 160	**↑**	**↓**
CIRBP	cold inducible RNA binding protein	**↓**	**↑**
HMGB3	high-mobility group box 3	**↓**	**↑**
WDR1	WD repeat domain 1	**↓**	**↑**

**Table 8:** The TFs with inverse activities of moderate AD and BC in grade Ⅱ.

TFs	Description	AD moderate	BC gradeⅡ
SSRP1	structure specific recognition protein 1	**↑**	**↓**
ZNF160	zinc finger protein 160	**↑**	**↓**
CIRBP	cold inducible RNA binding protein	**↓**	**↑**
LPP	LIM domain containing preferred translocation partner in lipoma	**↓**	**↑**
MATR3	martin 3	**↓**	**↑**

**Table 9:** The TFs with inverse activities of severe AD and BC in grade Ⅲ.

TFs	Description	AD severe	BC grade Ⅲ
CIRBP	cold inducible RNA binding protein	**↑**	**↓**
SSRP1	structure specific recognition protein 1	**↑**	**↓**
ZNF160	zinc finger protein 160	**↑**	**↓**
HMGB3	high-mobility group box 3	**↓**	**↑**

Table 7 shows that there are 6 out of 10 TFs are inversely associated at the initial stage of AD and BC, they are ASH1L, CFLAR, ZNF160, CIRBP, HMGB3 and WDR1. And 5 and 4 TFs are in opposite regulatory directions in the middle and terminal period of AD and BC respectively (Tables 8 and 9). Among them, CIRBP is a TF which have the ability to control cellular response upon confronting a variety of cellular stresses, mediate neuroinflammation and regulate MAPK, Wnt, apoptosis and many cancer-related signaling pathways. From Tables 7 to 9 we can see that it is inversely associated during the deterioration of AD and BC. ZNF160 is another important TF which is closely related to pathogen recognition and activation of innate immunity by cytokines. ZnF160 motifs recognize DNA sequences by binding to the major groove of DNA via a short alpha-helix, it can also bind to RNA and protein targets. From Tables 7 to 9 we can see that ZNF160 is inversely associated during different courses of these two diseases with the increasing in AD and declining in BC. Although the regulatory directions of these TFs are different in different states, even in different regions for the same disease, they play important roles in many immunological disorders including regulatory processes in inflammation, apoptosis, cellular response, innate and adaptive immune response. Considering the diverse changes in the different datasets of diseases, deep analysis is needed based on more numbers of microarray datasets and more detailed molecular biology research.

## Conclusions

Accumulating epidemiological evidence and meta-analysis data suggest that there is a strong inverse correlation between AD and cancer. This suggests that there are shared genes or biological pathways regulated by both AD and cancer with dramatically different directions. Some significant genes and signaling pathways play critical but opposing roles in both AD and BC, such as p53 and Pin1; the Wnt, ERK/MAPK, and UPS signaling pathways; and some biological processes associated with metabolic dysregulation, such as oxidative stress, DNA damage/repair, aerobic glycolysis, inflammation and cellular immunity. Convincing evidence suggests that both AD and cancer are age-related immune dysregulation diseases and that age plays a crucial role in their pathogenesis. We know that high-throughput DNA microarray datasets can successfully investigate hundreds of thousands of gene expression profiles simultaneously; the high-dimensional data are typically the regulatory results of a small set of TFs through an interacting network. However, high-throughput technologies that measure TF activities are not yet available on a genome-wide scale. Therefore, some statistical computational methods were applied in this study to deduce the biologically significant information and underlying transcriptional regulatory structure for the inverse regulatory mechanisms between AD and BC. Based on our current understanding of the innate and adaptive immune system in neurodegenerative diseases and cancer, we focused on the contribution of inverse TFs in the present study to determine the immune balance and pathogenesis of AD and BC.

To identify the significant differential genes shared in AD and BC, FastICA was first applied to microarray datasets from these two diseases. Our previous studies showed that as an unsupervised matrix decomposition technique, FastICA preceded PCA and NMF in capturing the potential biological processes via a statistically independent assumption. Second, NCA was performed to determine the activities of the shared TFs and regulatory influences on TGs because understanding dynamic TF regulation is a key component of understanding disease pathogenesis. Based on the NCA results, dynamic gene regulatory networks were reconstructed for AD and BC, from which the inverse regulatory activities of TFs were clearly revealed. There were 17 TF activities with regulatory control strength acting on 166 TGs. Among them, 10 significant TFs, including TFs: ASH1L, CFLAR, CIRBP, HMGB3, LPP, SMARCA4, SSRP1, WDR1 and ZNF160, displayed inverse regulatory activities between AD and BC, where the activities increased in AD and decreased in BC. Conversely, the activity of the TF MATR3 decreased in AD and increased in BC.

From the molecular biological analysis, we found that all 10 inversely associated TFs were closely related to cytokines and played important roles in the innate immunity, especially the adaptive immune response. For example, TFs, such as ASH1L, CFLAR, HMGB3, ZNF160 and MATR3, displayed opposite behaviors on some cytokines; inflammatory factors; nuclear and tumor factors, such as IL-2, IL-6, NF-κB, and Irf3; immunogenic nucleic acids; papillomavirus E7/E6; caspase-3/caspase-8; Toll-like receptor; and so on. Among them, the activities of ASH1L, CFLAR, HMGB3 and ZNF160 increased in AD and declined in BC; conversely, the activities of MATR3 decreased in AD and increased in BC. Furthermore, some typical biological pathways related to the adaptive immune response were revealed by the reconstructed transcriptional regulatory networks based on the NCA results. These immune-related biological processes included the Wnt and MAPK signaling pathways, apoptosis, myeloid differentiation, neuroinflammation, B-cell differentiation, T cell-mediated immune responses, pathogen recognition and activation of innate immunity by cytokines, which are regulated by the same TFs, including CIRBP, LPP, SMARCA4, SSRP1 and WDR1, in both AD and BC, but in different directions. The experiments on two additional AD and BC datasets with different grades also show that the inverse associations of these TFs exists in the whole process of the diseases and play important roles in the deterioration of diseases. We believe that uncovering the inverse associations of cytokines and adaptive immune response in our work will add significant contributions to diagnosis, immunotherapy and pathogenic discovery in both AD and BC. The transcriptional regulatory mechanisms for the inverse associations between AD and BC, as well as many other prevalent cancers based on immune dysregulation, will be investigated further in our future studies.
